# Robotic neurorehabilitation: a computational motor learning perspective

**DOI:** 10.1186/1743-0003-6-5

**Published:** 2009-02-25

**Authors:** Vincent S Huang, John W Krakauer

**Affiliations:** 1 Motor Performance Laboratory, Department of Neurology, The Neurological Institute, Columbia University College of Physicians and Surgeons, New York, New York, USA

## Abstract

Conventional neurorehabilitation appears to have little impact on impairment over and above that of spontaneous biological recovery. Robotic neurorehabilitation has the potential for a greater impact on impairment due to easy deployment, its applicability across of a wide range of motor impairment, its high measurement reliability, and the capacity to deliver high dosage and high intensity training protocols.

We first describe current knowledge of the natural history of arm recovery after stroke and of outcome prediction in individual patients. Rehabilitation strategies and outcome measures for impairment versus function are compared. The topics of dosage, intensity, and time of rehabilitation are then discussed.

Robots are particularly suitable for both rigorous testing and application of motor learning principles to neurorehabilitation. Computational motor control and learning principles derived from studies in healthy subjects are introduced in the context of robotic neurorehabilitation. Particular attention is paid to the idea of context, task generalization and training schedule. The assumptions that underlie the choice of both movement trajectory programmed into the robot and the degree of active participation required by subjects are examined. We consider rehabilitation as a general learning problem, and examine it from the perspective of theoretical learning frameworks such as supervised and unsupervised learning. We discuss the limitations of current robotic neurorehabilitation paradigms and suggest new research directions from the perspective of computational motor learning.

## Introduction

Every year in the United States, approximately 780,000 people suffer a new or recurrent stroke. The majority of people survive, but with long-term disability[1]. In 1999, more than 1.1 million American adults reported limitations in activities of daily living (ADLs) as a result of stroke. About 50% to 70% of stroke survivors regain functional independence eventually. However, up to 30% are permanently disabled with 20% requiring institutional care 3 months post-stroke. Among ischemic stroke survivors who were 65 years or older, 50% report some form of hemiparesis and 30% were unable to walk without assistance[1].

Hemiparesis is a blanket term that encompasses general weakness, motor control abnormalities, and spasticity. Given the prevalence of motor function loss in stroke survivors, it is surprising that in a 2005 survey of stroke survivors in 21 states and the District of Columbia, only 31% received outpatient rehabilitation[1]. Although advances in stroke rehabilitation have traditionally lagged behind those in acute stroke treatment and secondary prevention, the development of robotics and other new rehabilitation approaches indicate that this is changing. Robotic neurorehabilitation is attractive because of its potential for easy deployment, its applicability across of a wide range of motor impairment, and its high measurement reliability. Most importantly for the purposes of this review, robots will allow more rigorous testing and application of motor learning principles to neurorehabilitation. In this review we begin by describing the general challenges facing post-stroke rehabilitation, and then introduce motor learning principles within the context of robotic neurorehabilitation. We focus on rehabilitation on the upper limbs, but draw on motor control and learning examples for the lower limbs when instructive.

### The challenges facing post-stroke rehabilitation

Ideally, the rehabilitation strategy for any given patient would be planned based on a sound knowledge of what the natural history of their recovery would be in the presence or even in the absence of standard rehabilitation approaches. Unfortunately, we have not yet reached this point. Extant data suggest that the time course of recovery from hemiparesis after stroke varies considerably both across recovery measures and across patients[2]. Several critical questions arise when considering the time course of motor recovery from the perspective of neurorehabilitation: Should the focus of rehabilitation treatment depend on where in the time course of recovery a patient is and not just on severity of impairment? For example, if a patient has significant remaining impairment at 3 months should the emphasis then switch to compensatory approaches, assuming recovery from impairment reaches a plateau at approximately 3 months? Conversely; should compensatory strategies not be emphasized early after stroke because these might interfere with ongoing recovery from impairment?[3]. Does intense rehabilitation focused on impairment change the time course of recovery itself, with continuation in improvement beyond 3 months? Another question concerns the degree of inter-individual variability with respect to recovery – is the situation as heterogeneous as is assumed or are there predictable rules for recovery that apply to the majority of patients, albeit with important exceptions? Finally, do practice-related changes in the brain interact with spontaneous biological recovery in the first few months after stroke, and, if so, are these effects of learning distinct from those seen in the chronic phase?

#### Recovery from impairment versus functional compensation

When assessing the efficacy of rehabilitation interventions it is important to distinguish between measurements of impairment and measurements of functional performance. Functional tests assess task-specific improvements such as grasping a wooden block or pouring water from glass to glass. Examples of such scales include the Action Research Arm Test (ARAT)[4,5], the Jebsen-Taylor Hand Test[6], the Wolf Motor Function Test[7], and the 9 Hole Peg Test[8]. Other scales such as the Functional Independence Measure (FIM)[9,10] and the Barthel Index (BI) [11-13] measure the capacity of patients to perform activities of daily living (ADLs). However, because compensatory adjustments are possible in many everyday tasks (e.g, moving the trunk to compensate for reduced movement ability at the elbow and shoulder), an improvement measured with one of these scales will not necessarily reflect true recovery[14]. The most common clinical scale used to assess recovery from impairment is the Fugl-Meyer Motor Assessment (FMA), which tests motor function, sensory function, balance, the range of motion at joints and joint pain[15,16]. The motor and balance sections of FMA are most commonly reported in the literature. Multiple items are rated on a 0-to-2 point scale in each portion. In the motor portion, 66 possible points can be achieved with the upper limb items and 34 points with the lower limb items. The upper limb items rate the patient's ability to retract, elevate, abduct, adduct, flex, and extend at the shoulders, elbows, upper arms, forearms, wrists and fingers. Importantly, the motor portion of the FMA is resistant to compensatory strategies and has good inter-rater and test-retest reliability[16]. While correlations between functional and impairment scores [17-20] are quite good, it is nevertheless important to choose the appropriate measure when assessing the efficacy of an intervention. Indeed it has been suggested that recent clinical trials may have failed as a result of poor choices of outcome measurements rather than a lack of efficacy of the therapeutic agent[21]. Unfortunately, there are no standard procedures for choice of outcome measures post-stroke, which makes comparison between rehabilitation approaches very difficult.

#### Current observations on the time course of recovery

Longitudinal studies on impairment recovery suggest that only 33 to 70% of patients with stroke recover useful arm ability, and initial paresis severity remains the best predictor of arm function recovery 6 months [22-24]. However, the observation that over 86% of the variance in impairment at 6 months (as measured with the FMA) is explained by impairment level at 30 days suggests that rehabilitation has little impact on impairment in the intervening 5 months[21]. Functional improvements show a different time course. Arm function at 6 months measured with BI is best predicted by functional improvement in the first few weeks post stroke, with the first week explaining 56% of the outcome variance[25,26]. It is notable that functional ability at 6 months is not as well predicted as impairment, which suggests that rehabilitation in the intervening period has more impact on function than it does on impairment, possibly due to a focus on compensation strategies. Kwakkel and colleagues compared the time course of recovery in patients who received lower or upper limb rehabilitation with the time course in control patients who had their arm and leg immobilized with an air-splint for 30 minutes a day, 5 days per week for 20 weeks after stroke. [27,28]. At 6, 12 and 20 weeks post-stroke, the BI, ARAT and walking ability (functional ambulation categories) score were significantly better for the arm-training and leg-training group than for the control patients. At 26 weeks, however, only the difference in ARAT score remained significant. [26]. Thus, current rehabilitation programs appear to accelerate the rate of functional recovery but not its final level [26]. The time course of recovery beyond 6 months is not well characterized. Kwakkel and colleagues showed that 10 to 30% of patients showed further improvement or deterioration in ADL measures[28]. More over, there appears to be a limited, early window during which rehabilitation has the most long-term impact[26,27]. Ottenbacher and Janell reviewed 36 trials and found that the effectiveness of rehabilitation diminishes with increasing delay to initiation of therapy[29].

#### Variability in recovery and in response to rehabilitation

Scientific studies on human subjects often begin with the premise that as the number of test subjects increases, the distribution of the dependent variable approaches the Gaussian distribution. With healthy subjects, movements are usually stereotypical across subjects and studies, allowing the number of subjects to be limited. The underlying assumption is that normal, healthy subjects operate fairly similarly to each other in terms of planning and execution of movements. In contrast, for patient studies it is assumed that there is large inter-individual variability (broader Gaussian) in the capacity to recover [30]. We recently challenged this widespread assumption. In a study of 41 patients after first-time stroke, we found that clinical predictors; age, gender, infarct location, imaged infarct volume, time to reassessment, and acute upper extremity FMA motor score, were only able to explain 47% of the variance in recovery (measured as change in the FMA) at 3 months[30]. Interestingly, however, these clinical variables could explain 89% of the variance if some of the patients with severe initial impairment, identifiable as regression outliers, were excluded. Indeed, with the "outlier" subpopulation excluded, there was a positive, proportional relationship between the increase in FMA score after typical rehabilitation and maximal potential recovery (66 – initial FMA score). This study demonstrated that much of the reported variability in recovery after stroke results from the existence of sub-populations of patients who respond differently to injury rather than Gaussian-distributed inter-subject differences. Importantly, that so much variance in recovery at 3 months could be accounted for by an initial measure of impairment suggests that standard rehabilitation approaches in the acute and subacute period have minimal impact on impairment. Thus new approaches in the first three months after stroke are sorely needed. It is possible that the emphasis on compensatory strategies during acute rehabilitation may interfere with attempts to rehabilitate at the level of impairment[3]. It has been shown that functional electrical stimulation given in the first two weeks or over the first three months after stroke leads to improvement on the FMA scale[31,32]. While well-controlled clinical trials are currently lacking, it is possible that robotic therapy during the acute and sub-acute phase of stroke recovery could augment changes in impairment driven by spontaneous biological recovery processes and perhaps extend the impairment recovery period beyond three months.

Less is known about the factors that determine variability in response to therapy in patients with chronic stroke. If it is true that the majority of patients recover proportionally from impairment at three months and impairment reaches a plateau at 3 months[21], then it can be speculated that patients with chronic stroke who seek further treatment are made up of two populations: those with severe initial impairment who recovered to moderate levels of impairment and those who remained severely impaired[30]. Additional support for the idea that there are two distinct populations of patients with chronic stroke comes from the observation by Ferraro and colleagues that the amount of improvement in FMA after robotic neurorehabilitation was larger in patients with moderate impairment than in patients with severe impairment[33]. Subsequent studies of robotic therapy in patients with chronic stroke also report better results in patients with some degree of residual function before beginning therapy [34-36]. However, it remains unclear what the optimal training method is, or what the optimal training regimen should be, or even whether all patients with chronic stroke can benefit from additional rehabilitation training.

#### The promise of robotics

Robots provide both movement controllability and measurement reliability, which makes them ideal instruments to help neurologists and therapists address the challenges facing neurorehabilitation. In a recent review, Riener examined technical differences between several robotic devices[37]. These devices take the form of either an actuated robotic arm (i.e., a manipulandum) or joystick, or an actuated robotic suit that encloses the affected limb like an exoskeletal frame. The robots have sensors that record movement data such as position, velocity and joint torques. The robots also have actuators the enable them to move the subject's limb.

Robots allow for more precise measurement, in terms of movement kinematics and dynamics, of both initial impairment and of impairment changes in response to treatment. Not only does this measurement capability virtually eliminate the effect of inter-rater differences on outcome assessments, but also allows a biomechanical model to be used to perform inverse dynamic analysis on movement data to compute forces at joints[38]. Analogous to musical scores, some motor control studies have suggested that movements are planned as the combination of a relatively small number of muscle co-contraction patterns called synergies [39,40]. Component analysis techniques can be applied to both identify these patterns and observe any changes after rehabilitation training[41]. Stochastic perturbation (e.g., small random kicks) can be applied to the limb to estimate its impedance and control using system identification techniques[42,43].

Unlike conventional therapy, robotic manipulanda or exoskeletons can deliver training at a much higher dosage (i.e., number of practice movements) and/or intensity (i.e., number of movements per unit time) with hundreds if not thousands of repetitions in a single session. This dosage per unit time may be a critical factor in rehabilitation as animal data show that changes in synapse density in primary motor cortex occurs after 400 reaches but not 60[44,45].

Robotic neurorehabilitation studies have generally reported beneficial effects on impairment measures but have not proven effective with respect to functional outcomes. In a systematic review of eight robotic neurorehabilitation trials, Prange and colleagues concluded that robotic therapies led to long-term improvement in motor control by increasing speed, muscle activation patterns and movement selection, although no consistent benefit was found with ADL measures (e.g., FIM)[46]. In another systematic review of robotic neurorehabilitation, Kwakkel and colleagues also concluded that ADLs (e.g., FIM) did not significantly improve despite obvious improvement in impairment (e.g., FMA)[47]. One reason for these outcome measure-dependent results may be that functional assessment scales such as FIM are insensitive to improved performance at the level of impairment in the affected limb because they focus on the level of compensation. Conversely, increases in movement range and force as assessed by FMA may not have real-life relevance if they do not translate to an improvement in ADLs [34,48]. It may be necessary to employ more challenging impairment scales without the ceiling effect of the FMA[16] to improve correlations with functional scales. One such impairment scale, the Motor Status Score (MSS)[49], builds on the FMA and features more finely graded assessments over a larger range of upper limb motion. We speculate, however, that what is needed to see improvement in ADL measures is subsequent intense training in everyday tasks once impairment has been reduced to a specific level. This serial approach (i.e., focus on impairment first and then on function) might address the apparent paradox of a parallel dissociation between impairment and functional measures after a particular rehabilitative treatment. Finally, robotic neurorehabilitation has shown a beneficial effect on recovery from impairment in patients with chronic stroke [33,50]. These results are exciting because they imply that the 3 month impairment plateau may not represent an absolute upper limit.

Besides their applicability to rehabilitation, robots have also been used extensively to study motor learning in healthy subjects. The basic paradigm is to introduce a force perturbation that induces large trajectory errors to which the subjects must then adapt. This approach has allowed scientists to test several hypotheses about the computational mechanisms of motor control and motor learning[51]. However, relatively little attention has been paid to these basic science results in the rehabilitation literature. These results and their underlying principles, however, are likely to help us understand why robotic neurorehabilitation may be effective and what can be done to improve existing protocols.

### Robotic neurorehabilitation and computational motor learning principles

Motor control scientists define motor learning loosely, considering it a fuzzy term that encompasses motor adaptation, skill acquisition, and decision making[51]. Neurorehabilitation is based on two basic assumptions: that motor learning principles apply to motor recovery and that patients can learn. Robots provide the means to quantitatively test these two assumptions.

One of the fundamental principles of motor learning can be succinctly summarized by a cliché – practice makes perfect. Better performance is correlated with the time and amount of practice devoted to learning a particular skill[52]. In a systematic review on the role of intensity of practice on stroke rehabilitation, it was concluded that there is a dose-dependent relationship between acute and sub-acute post-stroke therapy and outcome[53]. The same review also concluded that training intensity did not have a significant impact on the outcome in patients with chronic stroke although it was noted that the number of well controlled trials (3) was low. Pilot studies in which the intensity of robotic neurorehabilitation was matched to the low dosage of conventional therapy did not find extra benefit for the robot [54], which strongly suggests that the benefits of robotic therapy come from the ability to deliver, through automated administration, therapy at dosages higher than is possible with conventional therapy.

#### Motor adaptation, internal models, and after-effects

Motor adaptation is a learner's reaction to a change in the environment. Empirically, it refers to a learner's incremental return to baseline performance in response to an environmental perturbation that causes performance errors. For example, a pair of eye glasses has the effect of magnifying or shrinking the visual field. Therefore there is a misalignment between the learner's sense of object position in visual space and body position in proprioceptive space. Often with a new pair of glasses, people feel disoriented. If the learner's vision is distorted by the glasses, when he reaches for a target he will miss it. Motor adaptation occurs when he learns from the visual error to realign his planned trajectory. Another example of motor adaptation is walking in waist-high water. Since the viscosity of the water causes resistance to motion, a person must adapt to this novel environment and produce more force to overcome the resistance to make the same movement that he could make on land[55].

An experimental situation that induces motor adaptation has the learner hold the end of a planar robotic arm and make reaching movements while the robot produces a perturbation force (force field) that scales with movement velocity and deflects the reach sideways[56]. A key finding of motor adaptation studies in healthy subjects is that when the environmental alteration is removed (e.g., switching off the force field) the learner's adapted state temporarily persists as if the environment was still in the last altered state. That is, subjects make a movement based on the prediction that the environment will be the same as they last experienced it. This motor *after-effect *demonstrates that the learner does not merely react to environmental changes but also anticipates the expected dynamics of the new environment and moves according to a new set of expectations. Therefore, motor adaptation appears to rely on an update in the internal representation (internal model) of the external environment.

Do patients with stroke adapt in the same way as healthy subjects? When making movements in a dynamically changing environment, healthy subjects make adaptive compensatory adjustments, partially countering the environmental changes. Healthy people make these adjustments on a movement-to-movement basis based on a short history of prior movements; with the largest weight placed on the latest movement[57,58]. Scheidt and Stoeckmann used the MIT-Manus to compare force field adaptation in post-stroke and healthy subjects. They found that the compensatory strategy utilized by the post-stroke group was the same as the healthy group but the influence of the movement error from trial *n *on trial *n+1 *was lower in patients with stroke[59]. These data imply that patients can indeed adapt in the same way as healthy subjects, even though it may take more practice trials.

The fact that patients can adapt to novel force fields suggests that it may be possible to manipulate the training environment so that after-effects resemble normal movements. Indeed, in a simple reaching task with a robotic arm, Patton and colleagues used force fields to exaggerate patients' baseline movement errors, which resulted in after-effects that resembled more normal movements[60,61]. Reisman and colleagues used the fact that after-effects are a general phenomenon and are not limited to upper limb movements in a split-belt treadmill study of thirteen patients with chronic hemiparesis who showed asymmetry in inter-limb co-ordination during normal over ground walking[62]. In the experiment, one belt sped up and the other slowed down. Healthy subjects adapted to this speed differential by making longer strides on the fast belt and shorter strides on the slow belt and showed after-effects when the change of belt speed was gradual. Analogous to the Patton study described above, patients exhibited after-effects that transiently improved the symmetry of their gait pattern.

#### Distinction between motor adaptation and skill learning

The improved movement patterns present as after-effects are evidence that some patients with stroke have the physical capacity to perform desired movements. However, as promising as it initially seems, error-induced after-effects are short-lived: In the arm reaching study, after-effects lasted for 30 to 60 movements (about 2 to 4 minutes) after 600 training movements (about 40 minutes)[60]. In the gait asymmetry study, after effects lasted for approximately 50 strides, after approximately 120 training strides[62].

Why do patients not arrive at the desired after-effect patterns on their own? It is possible that motor adaptation may be distinct from motor skill learning (e.g. learning to tie shoe laces, playing tennis). In motor adaptation tasks, people seem to understand how to make the intended movement but do not know how much movement to make. Skill learning, however, is more about learning how to make the intended movement in the first place. To illustrate this idea, imagine a hierarchy of movement control for an arbitrary skill. On the top level is the control policy that describes the skill requirement. One level down is the adaptive mechanism to compensate for changes in the operating condition of the skill task. At the lowest level is the spinal reflex control of movements. When there is a change in the operating condition (but the intended skill stays the same), the middle level of control compensates in order to fulfill the desired outcome of the top level control, i.e., motor adaptation. Now suppose there are two parallel hierarchies for two different skills, A and B. We perturb the operating conditions in B such that the resultant compensated movement plan for B overlaps with that of skill A without perturbation. It appears now that the learner is performing skill A very well. However, the motor plan generated by hierarchy B is for the control requirements of skill B, not A. Under this hypothetical motor learning architecture, it is not sufficient for patients with stroke to substitute appropriate skill control with after-effects of another skill. To truly perform skill A well in all operating conditions, the learner must acquire the top level control of A. If this hypothetical architecture is true, then patients will require extended training to acquire the highest level of control. With a high dosage of practice, robotic devices may increase the chance of patients re-discovering the appropriate skill and retaining it in the long term. Whereas motor adaptation, although quick, is rapidly forgotten.

#### End-effector versus exoskeletal robotic systems

Current upper-limb systems can be broadly grouped into two types: end-effector (e.g., MIT/IMT-Manus, MIME, GENTLE/s) and exoskeleton (e.g., ARMin, Pneu-WREX, RUPERT, REHAROB)[37,63-67]. With end-effector systems, subjects hold a manipulandum that experiences robot-imposed forces. All the forces and measurements are thus at a single interface, which has the advantage of easy set-up for patients of different body sizes. An analysis of three studies that used different kinds of end-effector system (MIT-Manus, ARM Guide, MIME) did not show a significant difference with respect to ADL measures (i.e., FIM score)[47]. However, comparisons at the level of impairment have not been made, nor between training in 2 versus 3 dimensions.

With exoskeletal systems, the limb is enclosed in an actuated robotic suit, which conforms to the configuration of the limb. While a subject's limb can be constrained with an end-effector system to specify one limb configuration (e.g., ARM Guide), the mechanical flexibility of the exoskeletal type allows full specification of limb configuration and for forces to be applied and measured independently at each joint. Exokeletal systems have the advantage that forces can be applied and measured independently at each joint. To date no studies have directly compared the two types of robotic system of system but differences can be expected based on motor control principles.

#### Generalization of learning within and across task contexts

An important question to ask is how much learning of one movement generalizes to another. For example, will skill at tennis generalize to skill at table tennis? From a computational perspective, full generalization could be considered the situation in which the relevant control parameters are common across two tasks and just their values need to be adjusted. This question has begun to be addressed formally in studies using adaptation paradigms (e.g, force field and rotation adaptation), which indicate that generalization can occur, to varying degrees, across the workspace, limb configurations, effectors, and tasks.

To test whether healthy subjects can generalize across the workspace within a given task context, we asked subjects to adapt to a rotation in a single direction centered on the hand. Typically in a visual rotation paradigm, visual feedback is rotated and set at a single angular offset at the beginning of the adaptation phase. Thus a misalignment between vision and the planned movement trajectory is introduced. Adaptation generalized to the same movement direction but with a new arm configuration[68]. Similarly, Baraduc and Wolpert asked healthy subjects to reach and point to a target from the same starting point using their index fingers but with different initial arm configurations. As we found, adaptation generalized across different arm configurations[69]. The implication of these results is that full specification of arm configuration in robotic therapy may not always be necessary. In our visual rotation study, we also tested for generalization across movement directions. We found that generalization fell to zero as the angular difference between the trained and the tested direction extended beyond 45 degrees[68]. Similar results have also been observed in reaching studies with force perturbations[70,71]. Taken together, these studies suggest that within-task generalization is broad in limb configuration space, and narrow in the visual space. In robotic neurorehabilitation, therefore, it may be important to train patients across several movement directions to fully learn a task.

Motor adaptation can also show varying degrees of generalization from one body part to another (i.e., effector). In a series of studies, Sainburg and colleagues found that training with clock-wise perturbations of dynamics in one arm can generalize to the other arm with counter-clockwise perturbations [72-75]. Similarly, Cricimagna-Hemminger and colleagues found that adaptation to force perturbation can transfer across upper limbs, but only from the dominant to the non-dominant arm[76]. For within-limb generalization, we found that adaptation to a visuomotor rotation during planar arm movements with the arm and shoulder transfers to movements of the wrist, but not vice versa[77]. Furthermore, prior wrist training with the visual feedback rotation blocked this transfer. These data suggest that generalization of motor adaptation depends on the effector, and the history of training in particular effector contexts.

How is context assigned in motor adaptation? This an open question; however, there is evidence that such assignment is dependent on how training is given. Let us re-consider the split-belt treadmill experiment introduced earlier. The gradualness of the change in the belt speed was important. When the belt speed was changed suddenly, both healthy subjects and patients with stroke did not exhibit after-effects[62]. Similarly, an incremental introduction of a visuomotor rotation promotes greater adaptation[78]. It has also been shown that the gradual adaptation procedure not only promotes larger and longer-lasting after-effects, it also induces inter-limb transfer[79,80]. Prism after-effects ordinarily only last for minutes in healthy subjects, but Rossetti and colleagues noted that after-effects in patients with neglect were significantly larger and lasted up to two hours[81]. It has been proposed that there may be multiple, concurrent processes that optimally adapt to environment changes on different time-scales[82,83]. When changes are sudden (e.g., holding a hammer), the learner's adaptation is reactive: it forms quickly at the first instance of the environmental change but also quickly fades away when the environment returns to baseline. When environmental changes are slow (e.g., growth of the body, chronic illness), the learner's adaptation is slow, but also more persistent. It has been suggested that the longer lasting after-effects, seen in patients with neglect and in subjects after incremental adaptation, arise because in both cases subjects are unaware of the external perturbation and so attribute errors as self-generated [79,84]. Thus gradual training may be a viable method to bias patients' attribution of error to self, and thereby prolong retention of adaptation.

Successful rehabilitation also requires that training generalize beyond the trained task. For example, training on task A at a rehabilitation center must lead to improvement at home in performance not just for task A but also generalize to similar but untrained tasks B, C, and D. In this case, the context is specified at the task level. It remains unclear whether results about generalization in healthy subjects using adaptation paradigms can inform with regard to generalization across task contexts for patients. One concern is that in adaptation paradigms the task goal is always the same – get the displayed cursor in the target. In contrast, learning to hold a cup may not make you turn a doorknob better. Similarly for gait, successful stationary walking patterns on the treadmill need to generalize to kinetic walking on the ground. Thus generalization results and concepts derived from adaptation experiments may not apply to skill and rehabilitation. This issue is critical for robotic neurorehabilitation, because it originated from adaptation studies, and because the robot itself can be seen as a tool external to the patient. There is evidence that suggests that monkeys, and likely humans, distinguish between body and object context. Graziano and colleagues showed that there are neurons in posterior parietal cortex sensitive to the visual position of the monkey's own arm and a fake arm prepared by a taxidermist, but not a similarly shaped rectangular box or an attended fruit[85]. Therefore the key question in rehabilitation using a robot is, do patients learn to adapt to a novel tool, or do they truly re-learn the control of their own arm?

Takahashi and colleagues found that improvements in grasp brought about through repetitive rehearsals with a robotic exoskeleton, did not generalize to improvements in supination/pronation of the hand despite an overall increase in motor gains in the proximal arm[86]. Similarly, Hidler, Hornby and colleagues found that robot-assisted locomotor training was not better than conventional therapy for patients with sub-acute[87] and chronic stroke[88]. One reason that skill generalization was not seen in the studies above, is that, analogous to the gradual versus abrupt adaptation studies mentioned previously[80], patients may have learned to associate training-related changes with the robot rather than with their own body. Shadmehr and Krakauer have recently suggested in a review of lesion studies that the function of the cerebellum is system identification to facilitate error-driven motor adaptation[89,90] but that other anatomical regions are needed to form sensory beliefs, calculate the cost and rewards of actions, and compute control policies. Adaptation alone may not be sufficient because skill learning is likely to require all of these computations.

#### Effects of training schedule on motor learning

Varying training schedules may also have a positive effect on rehabilitation. It is known that when training sessions are temporally distributed over a period of time, the retention of performance is better than if the training sessions are massed together[91,92]. It is postulated that the minutes and hours between training sessions may allow consolidation of motor memories [93-97]. More recently, it has been shown that intervals between movements, even of a few seconds, can benefit motor learning by promoting spatial generalization on a trial-to-trial basis[98]. Robot rehabilitation is an ideal vehicle for automated delivery of pre-specified training schedules.

Training is only meaningful if the acquired motor skill is retained beyond the training sessions. A motor learning principle related to scheduling and retention of learned motor skills is contextual interference. Instead of training on only one task per session, mixing several tasks in one session has been shown to produce better retention. Indeed, this is even the case when the performance of an individual skill is better at the end of a block during which it is the sole skill practiced. Shea and Morgan asked healthy subjects to practice three different punching styles, and tested their performance at 10 minutes and at 10 days after training. They found that people who inter-mixed trainings retained their learned skill better than those who practiced one punch style at a time[99]. Similar results on the acquisition and retention of motor skills were demonstrated in basketball shooting[100] and in functional movement learning after stroke[101,102]. It is suggested that an inter-mixed schedule may aid learning because the variability in training skills promotes the learning of each individual skill as part of an overall problem to be solved, the brain solves a problem in each trial rather than just replaying a movement from memory.

#### Degree of subject participation in robotic training

The most common robot rehabilitation protocols employed to date involve one or a combination of the following: 1) the robot initiates the movement and produces an assistive force to push the subject's arm with a pre-defined trajectory and speed; 2) the subject initiates the movement but the robot then produces an assistive force; 3) the subject initiates and pushes the robot to an intended target while the robot provide a resistive force; 4) the subject initiates and pushes the robot to an intended target; the robot only corrects if the movement is off course or too slow. Most studies have not distinguished the effects of the these protocols since participants usually receive a mixture of the three either as a fixed paradigm or at the discretion of the study therapist[46]. Attempts to determine the relative efficacy of these protocols have been inconclusive [103,104].

These protocols can be implemented with an impedance controller (e.g., IMT-Manus) or an admittance controller (e.g., GENTLE/S). To visualize how an impedance controller works, imagine a ball-bearing representing hand position at the bottom of a symmetrical concave well. The slope of the well wall provides the impedance that keeps the bearing at the center of the well. If the slope becomes steeper (i.e., higher stiffness) in one direction +x (i.e., higher stiffness) and flat (i.e., zero stiffness) in the opposite direction -x, the bearing will move toward the -x direction. The shapes of the well can be modified such that the bearing encounters a low level of impedance in the direction of desired trajectory and a high level of impedance in any other direction. In engineering terms, the impedance controller reacts to a displacement with a restoring force. With an admittance controller, the system measures the amount of the push by the subject, and reacts with a displacement. In engineering terms, the admittance controller reacts to a force with a restoring displacement. Thus, the impedance controller has a high gain simulating a stiff wall, and a 0 gain simulating free air; the admittance controller has a high gain simulating free air, and a 0 gain simulating a stiff wall. An overly high gain can lead to over-compensation and oscillations of the robot when there is a small difference between the actual system state and the desired state.

The haptic interface between human and the robot may promote different motor control strategies. The choice of a particular motor control strategy may depend on an innate cost function that weights rewards (e.g., speed, accuracy) and costs (e.g., pain, effort)[105,106]. For example, the patient's may not even be able initiate movement without assistive force. On the other hand, the patient may exert less force when he or she knows the rehabilitating robot will provide assistance. To answer this, imagine that over the course of a movement, the patient's motor controller produces a continuously modulated motor command. Reinkensmeyer and colleagues hypothesized that the continuous output of the patient's motor controller is reduced by an amount proportional to the controller's previous output[107]. In other words, the human real-time motor controller has memory. As a result, patients' force output exponentially decays over the course of a movement allowing the initial momentum to carry over into the later phase of movement, and the robot's memory-less assistive force controller takes over the performance. By adding memory to the robot controller (via the addition of a slack term that depends on the previous robot output), they found they could increase the patients' share of load, therefore increase their active participation in the movement exercise.

#### Desired movements

What exactly are patients learning with robotic neurorehabilitation? To begin to answer this question, it is useful to borrow ideas from machine learning. The field of machine learning examines the effectiveness of training algorithms. One type of learning algorithm is called supervised learning, in which the learning agent is given pre-determined training examples with their corresponding desired solutions. The learning agent is provided with an algorithm to generate a function that maps the training inputs to the provided desired outputs. In the implementation of many robot rehabilitation systems, patients undergo supervised learning as the desired trajectories are pre-determined. The control software of the robot then attempts to minimize the spatiotemporal difference between the actual position of the affected limb and the desired trajectory.

How are desired trajectories determined? Early motor control studies provide clues. In the 1980s, motor control scientists debated whether reach plans were encoded in joint coordinates (joint angles at the shoulders and elbows) or visual coordinates (i.e., Cartesian coordinates centered on the eyes, head or hand). The latter is suggested by studies showing that natural arm movements are quite stereotyped across the population: hand paths are straight and the velocity profiles are smooth and bell-shaped [108], even in congenitally blind people[109]. Hogan and Flash [110] found that they could mathematically represent the smoothness of hand motion by the solution function that minimizes the third time derivative of position (i.e, jerk). The minimum jerk trajectory is attractive because it only requires one to know the length and desired duration of a movement to completely describe the trajectory with an analytic function of time. Systems such as the IMT-Manus and the ARM Guide are programmed with the minimum-jerk trajectory[111,112].

The applicability of the smooth path described by the minimum-jerk trajectory is, however, limited when we consider the complexity of most ADLs, for example, reaching for an object around an obstacle. In fact, depending on the goal of the reach, the hand trajectory may not conform to that of the minimum jerk trajectory. Nathan and Johnson examined functionally oriented reach-to-grasp movements with different objects[113]. In the study, subjects were asked to reach for a comb, retrieve the comb, brush twice, and return the comb. They found that the reaching portion of the movements were significantly different from minimum jerk predictions.

One reason for this deviation from minimum-jerk trajectory is that additional goal-dependent criteria also need to be met for any given movement. Optimal feedback control theories formally address this idea. Under the optimal control scheme, there is a cost function that includes task-relevant parameters such as the goal of the movement, the need to avoid obstacles and the effort required [114]. The notion of optimal feedback control also means that people do not adapt by simply trying to cancel out perturbations in the environment in order to arrive back at a pre-perturbation desired trajectory. Rather, adaptation is a re-optimization process that plans a new desired trajectory based on the new environment[115].

In addition to minimum-jerk and optimal feedback control trajectories, other smoothness criteria have also been used. For example, the NeReBot system uses cubic-spline interpolation of pre-defined steps of arm configurations to obtain a smooth desired trajectory[116]. Another strategy to implement a reference trajectory is to derive it from the movement of the unaffected arm accomplishing the same goal. The Mirror Image Movement Enabler (MIME) system is a 6-degree-of-freedom industrial end-effector type robot that allows for uni- and bi-manual movements that employs this strategy as one of its possible operating protocols[50]. However, the MIME pilot study employed multiple operating protocols concurrently so it is not clear whether the protocol is better than the others. In summary, a critical and as-of-yet unresolved question with regard to training trajectory is whether the therapist should choose an invariant optimal trajectory (e.g., as seen in healthy subjects or the unaffected arm) or assume an invariant cost function and allow for trajectory re-optimization.

#### Training away compensation

One reason that patients develop compensatory strategies to accomplish functional tasks is that these strategies were perhaps indeed optimal for their level of impairment in the sub-acute period. However, compensatory strategies can become a habit that is hard to break if brain function recovers to a point where a more normal movement would be attainable with practice. In other words, a patient's performance may become stuck in a local optimum. It may be possible to change the environmental condition for the learner such that the local optimum disappears and thereby encourages exploration for the global optimum. A very simple example of this is provided by constrained-induced movement therapy (CIMT). In CIMT, the non-affected arm is constrained, therefore a local minimum, use of the unaffected arm, is eliminated and patients are thereby obligated to explore command space with their affected arm. Robotic neurorehabilitation, through the application of resistive, assistive and other forces, is ideal to promote exploration of movement strategies because the difficulty level of the robotic therapy can be titrated to the patients' impairment level to promote unlearning of compensatory habits and reduction in impairment. In other words, the cost function can be artificially titrated to promote plasticity. Robotic neurorehabilitation in the acute and sub-acute phases might even pre-empt patients from becoming stuck in a local optimum. For example, conventional contextual interference paradigms, as described previously, randomize equal proportions of training trials for individual tasks to enhance overall skill retention compared to block training design. Choi and colleagues recently asked whether the benefit of contextual interference can be further improved by a varying schedule that takes into account the skill level of the learner and the difficulty of the individual tasks[117]. Healthy subjects were asked to adapt to four visuomotor rotations in 3 consecutive daily sessions, each made up of 120 pseudo-randomized trials. An algorithm that adaptively increased the proportion of training trials for the worse-performed rotation was used in each session. Subjects' performance was shown to be better with the adaptive schedule than with a uniformly distributed schedule in a delayed retention test. Choi and colleagues then adaptively allowed more or less time for each movement based on an on-line estimate of performance, and showed that this also led to better retention.

#### Supervised versus unsupervised learning in motor learning

Most of the experimental motor learning literature is concerned with supervised or passive learning because the tasks involve simple or stereotypical movements and the desired trajectories are obvious. Subjects are commonly instructed: "make a straight reaching movement to the target". However, a task may require more than a simple, stereotypical movement to accomplish. If the ability to make a complete movement is lost after brain injury, how does one re-learn the movement when it may be accomplished in many different ways through many different commands, and no instruction is given as to how to proceed? Active learning is the study of algorithms that select teaching examples for use in supervised learning. Cohn and colleagues studied this theoretical problem and developed an optimal algorithm based on the learner's uncertainty about various components of the task[118]. It was proposed that the best sequence of actions the learner should choose is one that minimizes the learner's uncertainty about the overall task. We recently tested this theoretical framework with a force-field adaptation paradigm in healthy subjects. We found that when given a choice of which target to visit, subjects would repeat those actions that had resulted in large errors, which supported the uncertainty hypothesis. However, people also repeated actions that were already perfect, taking time away from learning other less accurate actions, thereby making their choice of target sequence inefficient[119]. The implication of the study is that it might be possible to improve motor learning with robotic neurorehabilitation by imposition of an optimal, supervised sequence.

Unlike supervised learning, in unsupervised learning the learner does not know the desired outcome. Instead, the learner interacts with the environment to extract information and arrives at an optimal solution [120,121]. One can imagine a situation where a child fumbles around to learn to play with a novel toy. By pressing, stretching or simply re-orienting the toy, he receives proprioceptive, tactile, audio or visual feedback and may eventually discover a magic button that makes the toy play music. Similarly in motor learning, one can adjust and try different actions until the optimal set of parameters are found. Unlike supervised learning, "errors" are not explicitly corrected in unsupervised learning. Theoretical investigations on this learning principle are most studied in a sub-field of machine learning called reinforcement learning, in which the learning focuses on reinforcement of actions that result in better performance. Reinforcement learning may apply to robot-assisted rehabilitation. For example, a recent study of robotic therapy for the hand employed two types of training modes. In the active assist mode, patients initiated a grasp movement and the robot helped the completion of the grasp if the movement was not completed within a few seconds. In the active non-assist mode, patients attempted the grasp without robot assistance. It was found that active assist mode was better[86]. In the reinforcement learning framework, the successful completion of the movement with robot assistance may have helped to identify the desired sensory states and their transitions, which subsequently helped to identify the motor commands required to achieve these state transitions.

The efficacy of the learning interactions depends on the sequence of actions the learner chooses. Should a patient keep practicing a particular movement with the hope that he can build on his previous experience and will eventually "get it" (i.e., exploitation of current knowledge) or should he try other movements that may lead to the correct movements (i.e., exploration of unknowns)? Careful assessment of a patient's trial-to-trial performance should make it possible to titrate robotic assistance in order to shift their exploitation-exploration tradeoff. Current robotic neurorehabilitation paradigms are almost certainly combining supervised and unsupervised learning principles, even if this is not the explicit intention. Determining the relative benefit of one learning principle over another and the optimal balance between them will require further investigation.

## Conclusion

Computational motor learning principles provide a framework for the design of optimal rehabilitation protocols. Since motor impairment is the common denominator of all functional motor disabilities, we suggest that in acute and sub-acute stages of recovery that it would be more effective to focus rehabilitation efforts on restoration of impairment and avoid a premature emphasis on compensation. In order for rehabilitation to have an impact at the impairment level, high intensity (i.e., dosage per unit time), high dosage, and realistic movement training in 3-dimensions will most likely be required. After a given level of impairment improvement, therapy would transition to an emphasis on functional ability. In patients with chronic stroke, outcome prediction tools need to be developed to assess further potential for further reduction in impairment. A comprehensive rehabilitation program, therefore, may require therapy protocols and equipment that differ in the acute and chronic stages of recovery. The capacity of robots to deliver training with high intensity, dosage, reliability, repeatability, quantifiability, and flexibility makes them an ideal tool to both test, and eventually implement rehabilitation paradigms to aid motor recovery from stroke and other forms of brain injury and disease.

## Competing interests

JWK declares that he has no competing interests. VSH has a pending consulting agreement with Motorika, a company that manufactures robotic technology for the purpose of rehabilitation.

## Authors' contributions

VSH formulated concepts and ideas and drafted the manuscript. JWK contributed concepts and edited and revised the manuscript. Both authors read and approved the final manuscript.
